# Evaluation of Douyin Short Videos on Mammography in China: Quality and Reliability Analysis

**DOI:** 10.2196/59483

**Published:** 2025-02-19

**Authors:** Hongwu Yang, Chuangying Zhu, Chunyan Zhou, Ruibin Huang, Lipeng Huang, Peifen Chen, Shanshan Zhu, Huanpeng Wang, Chunmin Zhu

**Affiliations:** 1 Department of Radiology of First Affiliated Hospital, Shantou University Medical College, Shantou, China

**Keywords:** breast cancer, mammography, Douyin, information quality, social media, video, DISCERN, Global Quality Score, web-based education, cancer screening, health information, medical content

## Abstract

**Background:**

Breast cancer is the most common malignant tumor and the fifth leading cause of cancer death worldwide, imposing a significant disease burden in China. Mammography is a key method for breast cancer screening, particularly for early diagnosis. Douyin, a popular social media platform, is increasingly used for sharing health information, but the quality and reliability of mammography-related videos remain unexamined.

**Objective:**

This study aimed to evaluate the information quality and reliability of mammography videos on Douyin.

**Methods:**

In October 2023, a search using the Chinese keywords for “mammography” and “mammography screening” was conducted on Douyin. From 200 retrieved videos, 136 mammography-related videos were selected for analysis. Basic video information, content, and sources were extracted. Video content was assessed for comprehensiveness across 7 categories: conception, examination process, applicable objects, precautions, combined examinations, advantages, and report. Completeness was evaluated using a researcher-developed checklist, while reliability and quality were measured using 2 modified DISCERN (mDISCERN) tool and the Global Quality Score (GQS). Correlations between video quality and characteristics were also examined.

**Results:**

Among the video sources, 82.4% (112/136) were attributed to health professionals, and 17.6% (24/136) were attributed to nonprofessionals. Among health professionals, only 1 was a radiologist. Overall, 77.2% (105/136) of the videos had useful information about mammography. Among the useful videos, the advantages of mammography were the most frequently covered topic (53/105, 50.5%). Median values for the mDISCERN and GQS evaluations across all videos stood at 2.5 (IQR 1.63‐3) and 2 (IQR 1‐2), respectively. Within the subgroup assessment, the median mDISCERN score among the useful and professional groups stood at 2 (IQR 2‐3) and 3 (IQR 2‐3), respectively, surpassing the corresponding score for the unhelpful and nonprofessional groups at 0 (IQR 0‐0) and 0 (IQR 0‐0.75; *P*<.001). Likewise, the median GQS among the useful and professional groups was evaluated at 2 (IQR 1.5‐2) and 2 (IQR 1‐2), respectively, eclipsing that of the unhelpful and nonprofessional groups at 1 (IQR 1‐1) and 1 (IQR 1‐1.37; *P*<.001). The GQS was weak and negatively correlated with the number of likes (*r*=−0.24; *P*=.004), comments (*r*=−0.29; *P*<.001), and saves (*r*=−0.20; *P*=.02). The mDISCERN score was weak and negatively correlated with the number of likes (*r*=−0.26; *P*=.002), comments (*r*=−0.36; *P*<.001), saves (*r*=−0.22; *P*=.009), and shares (*r*=−0.18; *P*=.03).

**Conclusions:**

The overall quality of mammography videos on Douyin is suboptimal, with most content uploaded by clinicians rather than radiologists. Radiologists should be encouraged to create accurate and informative videos to better educate patients. As Douyin grows as a health information platform, stricter publishing standards are needed to enhance the quality of medical content.

## Introduction

Breast cancer is the second most common cancer and the fourth leading cause of cancer death worldwide. In 2022, an estimated 2.3 million new cases (11.6% of all cancer cases) were diagnosed, and 666,000 deaths (6.9% of all cancer deaths) occurred, and the number of new breast cancer cases is projected to reach 4.4 million by 2070 [1]. Among women, breast cancer is the most commonly diagnosed cancer, and it is the leading cause of cancer deaths globally. In 2022, breast cancer accounted for approximately 15.4% of all deaths in global female patients and 6.9% of all cancer deaths [2]. As the second most common cancer in Chinese women, an estimated number of 357,200 new cases of breast cancer occurred in 2022, accounting for approximately 7.4% of total new cancer cases in China and 15.5% of global breast cancer cases [3]. The World Health Organization recently launched the Global Breast Cancer Initiative with the aim of reducing breast cancer mortality by fostering timely diagnosis and adequate treatment and patient management [4]. The 5-year survival rate in patients with early breast cancer is very high; thus, early screening, detection, and treatment are important [5]. Measures used for breast cancer screening in the “Guidelines for breast cancer diagnosis and treatment by China Anti-Cancer Association (2024 edition)” include mammography, ultrasonography, clinical breast examination, breast self-examination, and magnetic resonance imaging. Guidelines recommend that the starting age for breast cancer screening in the general risk population is 40 years. However, for people at high risk of breast cancer, the start of screening may be earlier than the age of 40 years. For those older than 70 years of age may consider opportunistic screening [6]. Mammography is one of the most effective methods for breast cancer screening, especially for early breast cancer diagnosis, and it has been a major contributor to the decline in breast cancer mortality rates [7-9]. At present, there is no nationwide screening program for breast cancer in China. A cross-sectional survey conducted with a convenience sample of 494 Chinese women indicated that participation in screening practices ranged from 27.5% for BSE, 36.4% for clinical breast examination, 23.5% for mammography, and 40% for ultrasonography [10].

With the widespread adoption of internet technology, web-based platforms have become a primary channel for accessing public information. As of June 2023, China’s internet user base has expanded to 1.079 billion, with short video users reaching 1.026 billion, representing 95.2% of the total internet population [11]. The short video format has emerged as a dominant force in the new media landscape, due to its low barrier to entry, concise format, and rapid dissemination capabilities, making it one of the most preferred mediums for health information acquisition. While TikTok stands as a global social media giant, operating in over 160 countries with more than 1 billion monthly active users [12], its services are unavailable in China due to internet regulations. Instead, Douyin (the Chinese equivalent of TikTok, literally meaning “shaking sound”) has established itself as a national phenomenon, boasting over 750 million daily active users and ranking among the country’s most popular applications [13]. The platform’s influence on health communication is particularly noteworthy. The Douyin Health Science Data Report indicates that daily health science content reaches more than 200 million users as of March 2023 [14]. This trend is further supported by data from the 2023 Douyin Health Lifestyle New Paradigm White Paper, which reveals that during the first half of 2023, the platform hosted more than 10 million creators specializing in health care knowledge content [15]. Notably, industry reports highlight that 42% of Douyin’s user base comprises individuals aged 40 years and older [16], suggesting a significant engagement of mature audiences with health-related content on the platform.

Mammography screening often evokes feelings of anxiety and discomfort among patients, prompting many to seek preparatory information and clarification through social media platforms. High-quality educational videos can serve as valuable resources in this context, potentially contributing to improved health outcomes. Research evidence underscores the effectiveness of video interventions in promoting mammography screening. A study focusing on Chinese immigrant women demonstrated that culturally adapted videos, developed based on the health belief model, significantly enhanced screening intentions, breast cancer knowledge, risk perception, and understanding of mammography benefits [17]. Furthermore, empirical evidence indicates that brief preprocedure video interventions can substantially increase both physician referrals for screening mammography and patient compliance with screening completion [18]. These findings highlight the potential of video-based educational tools in addressing patient concerns and facilitating informed decision-making regarding breast cancer screening.

Despite the growing reliance on social media for health information, significant challenges persist regarding the reliability and accuracy of such content. The diverse backgrounds of content creators and viewers, coupled with the absence of robust verification mechanisms, make it difficult to assess the quality and credibility of health-related information on these platforms [19]. A comprehensive systematic review of reviews revealed that the prevalence of health misinformation on social media ranges from 0.2% to 28.8% [20], posing substantial risks to users. Exposure to inaccurate health information through videos may lead to severe consequences, including delays in seeking appropriate care or even life-threatening situations [21,22]. Previous research has extensively evaluated the quality of health-related content on traditional video-sharing platforms like YouTube and TikTok, covering various medical topics such as cervical spondylosis, gastroesophageal reflux disease, and broken heart syndrome [23-25]. However, the examination of mammography-related video content remains limited. To date, only 2 studies have assessed the quality of mammography videos on YouTube [26,27], both of which identified inconsistencies in the quality of information presented. Notably, no studies have yet evaluated mammography-related content on Douyin, the Chinese counterpart of TikTok. This research gap underscores the need for systematic evaluation of mammography-related short videos on Douyin, particularly considering its massive user base in China. Therefore, this study aims to comprehensively assess the quality and reliability of mammography-related short videos on Douyin by analyzing their characteristics, sources, and content.

## Methods

### Search Strategy

To minimize the bias introduced by personalized recommendation algorithms, we used 3 tactics: creating a new Douyin account specifically for evaluation, disabling Douyin’s personalized recommendations to eliminate differential content recommendations caused by user habits, and banning access to mobile location services. All videos were viewed without any actions such as downloading, liking, commenting, collecting, or sharing. Evaluation tasks were carried out by 2 qualified radiologists (Chuangying Zhu and HY) from the division of radiology in a tertiary teaching hospital.

The keywords “钼靶” (“mammography” in Chinese) and “钼靶检查” (“mammography screening” in Chinese) were searched in the Douyin app on October 22, 2023, with no limits placed on the release time. Douyin offers 3 ways to filter videos: overall ranking, most recent, and most likes. We used the overall ranking mode to retrieve the top 100 videos because most consumers use this default sorting option. We chose the threshold number of 100 for 2 reasons. First, Douyin’s search function takes topic relevance into account; the most relevant mammography videos tend to appear at the top of the results list, and it is difficult to observe any pertinent videos when the results exceed 100. Second, most general health users apply the “least effort” principle when searching for information on the web; they tend to concentrate on the top search results. In this study, we included videos directly related to mammography. The exclusion criteria were videos not in Chinese, videos not related to mammography, duplicate videos, videos shorter than 10 seconds in length, and videos that were unavailable.

### Data Collection

A Microsoft Excel spreadsheet was created by a researcher for data collection. Video information analyzed in this study was the identity of the uploader; the duration in seconds; the number of “likes” as indicated by the heart icon; the number of comments, saves, and shares the video received; and the number of days since the video was uploaded.

We divided the videos into 2 main groups according to whether the uploaders were professional or nonprofessional. Professional videos consisted of videos uploaded directly by board-certified physicians, health channels, and hospital channels. Most health channels and hospital videos were narrated by doctors. Nonprofessional videos included those uploaded by patients and other individuals.

### Quality and Reliability Assessment

The quality and reliability of the video were evaluated based on the following criteria: the accuracy and comprehensiveness of the content, the clarity and fluency of information delivery, and the overall usefulness of the video to its intended audience.

No validated tools for assessing mammography video content are available in the literature. According to the American Cancer Society recommendations for the early detection of breast cancer [28] and the China Anti-Cancer Association Breast Cancer Diagnosis and Treatment Guide and Standard (2024 Edition) [6], 2 qualified radiologists (Chunmin Zhu and RH) from the division of radiology in a tertiary teaching hospital, with more than 10 years of experience in the radiological profession, developed a completeness checklist to assess the quality of mammography video content (Table 1). The 7 categories cover most aspects of mammography: conception, examination process, applicable objects, precautions, combined other examinations, advantages, and report. A video was awarded 1 point in each domain if it mentioned the content listed in Table 1, resulting in a final score ranging from 0 to 11. A score of 0 indicated that there was no accurate content in any of the 7 earlier-mentioned areas of mammography, whereas a score of 11 indicated that a video contained accurate information in all areas. Videos were then further categorized as useful or unhelpful according to the final score. Videos with a score of 0 were considered unhelpful if they only dealt with personal experiences or testimonies without providing any scientific content, whereas useful videos received a score of ≥1. We used the modified DISCERN (mDISCERN) tool and the Global Quality Score (GQS), previously used in many studies of Douyin, as instruments to assess the quality of information in each video.

**Table 1.** Completeness checklist.

Content	Description
Conception	1. Basic principles: mention that a mammogram is done with a machine designed to look only at breast tissue, with low-dose x-rays.2. Radiation: mention that mammogram exposes the breasts to small amounts of radiation.
Examination process	3. Remove upper body clothing: mention that the patient must remove clothing above the waist to have a mammogram.4. Pain: mention that it might feel some discomfort when the breasts are compressed, and for some women, it can be painful.5. Two positions for unilateral breast: mention that x-ray pictures of each breast are taken, typically from 2 different angles.
Applicable objects	6. Age: mention when to start a mammogram and how often.7. High risk: mention that women who are at high risk for breast cancer based on certain factors should get a mammogram every year, typically starting before 40 years of age.
Precautions	8. Special period: mention that women who are in a special period, such as preparing for pregnancy, pregnant, or breastfeeding, and those who have undergone breast augmentation surgery need to inform doctor in advance. Mammograms are generally not recommended for pregnant women. It is best to schedule the examination about a week after her period.
Combined other examinations	9. Mention that breast ultrasound and magnetic resonance imaging can help find some breast cancers that cannot be seen on mammograms.
Advantage	10. Mention that mammograms have a great advantage in detecting calcifications.
Report	11. Mention that what is the breast imaging reporting and data system.

The DISCERN criteria are a validated scoring system developed by an Oxford University research team to assess the information quality and reliability of content related to consumer health information on treatment options [29]. The mDISCERN tool was modified by Singh et al [30] and is based on a 5-point Likert scale that examines goals, reliability of information sources, bias, areas of uncertainty, and additional sources. According to the mDISCERN score, the reliability of video content is considered good for a DISCERN score of >3 points, moderate for a DISCERN score of 3 points, and poor for a DISCERN score of <3 points.

The GQS, which was developed by Bernard et al [31], is a 5-point Likert scale used to assess the quality of a video based on the flow of information, completeness of the information presented on a particular topic, and usefulness of information to patients. A GQS of 1 is considered very poor, 2 is considered poor, 3 is considered fair, 4 is considered good, and 5 is considered excellent. The detailed information for mDISCERN and GQS is available on the web as in MultimediaAppendices 1  2.

Before starting to score the videos, radiologists first reviewed the official DISCERN and GQS instructions and referred to a simplified Chinese version [32], the latter is more adapted to the Chinese language and culture. To ensure consistency, prescoring discussions were mandatory. After reaching a consensus on the first 20 videos, the evaluators independently reviewed the subsequent entries. The original scores of the 2 radiologists (C Zhou and HY) were independently recorded. The scores of mDISCERN and GQS given by the 2 researchers (C Zhou and HY) were averaged to obtain an overall score, which was then used in the analysis. Any disagreements about the completeness checklist were resolved by consensus.

### Statistical Analysis

SPSS (version 27.0; IBM Corp) was used for data entry and analysis. Data are summarized as frequency (n) and percentage (%) for categorical variables and median (IQR) for ordinal variables. The normality of the data was analyzed using the Shapiro-Wilk test. Because the data were not normally distributed, the Mann-Whitney *U* test was used to compare the continuous variables between the 2 groups. Cronbach α coefficients were used to calculate the agreement between the 2 researchers. Spearman correlation tests were used to assess relationships between parameters. The correlations were interpreted based on the magnitude of the Spearman correlation coefficient (*r*), with the following thresholds used as a guide to describe the strength of the relationships: *r*<0.1 is considered a negligible correlation, 0.1≤*r*<0.4 is a weak correlation, 0.4≤ *r*<0.7 is a moderate correlation, 0.7≤*r*< 0.9 is a strong correlation, and *r*≥0.9 is a very strong correlation. These thresholds were adapted from conventional guidelines for interpreting correlation coefficients, as discussed in the literature [33]. Differences were considered statistically significant at a *P* value of <.05.

### Ethical Considerations

No clinical data, human specimens, or laboratory animals were involved in this study. All information used in this study was obtained from publicly released Douyin videos, and none of the data involved personal privacy. In addition, the study did not involve any interaction with users; therefore, no ethics review was required. All data were deidentified, and no individual users, videos, or screenshots are identifiable in this manuscript or its supplementary materials.

## Results

### Video Selection Process

**Figure 1.**
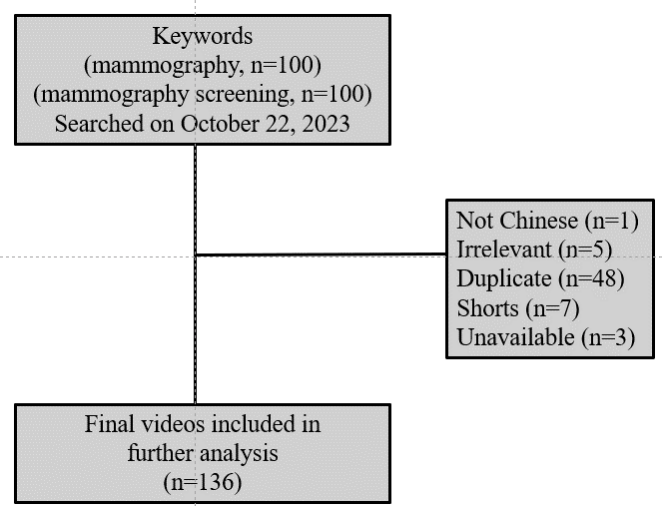
Flowchart of videos included in the study.

In total, 200 videos were screened, and 136 were included in the study. The 64 excluded videos were 1 video in a non-Chinese language, 5 irrelevant videos, 48 duplicate videos, 7 short videos, and 3 unavailable videos (Figure 1).

### Video Characteristics

The statistical analysis showed that the mammography videos ranged from 11 to 876 seconds. At the time of this study, the 136 short mammography videos had received 1,788,786 likes, 288,802 comments, 110,224 saves, and 598,393 shares. Each short video received 2 to 256,000 likes, 0 to 81,000 comments, 0 to 21,000 saves, and 0 to 145,000 shares. The most recent video was posted 21 days before the data collection, while the oldest had been on Douyin for more than 3 years. The median duration of the videos was 49.5 (IQR 32.5‐76.75) seconds; the median indicators of engagement comprised 414 (IQR 155.75‐1887.25) likes, 50.5 (IQR 20‐286.25) comments, 56 (IQR 19-201.75) saves, and 61.5 (IQR 12‐275.75) shares; and the median time since upload was 382.5 (IQR 116.25‐635.75) days. The characteristics of the included videos are shown in Table 2.

**Table 2.** Characteristics of videos about mammography on Douyin.

Characteristics	Median (IQR)	Range
Duration (seconds)	49.50 (32.50‐76.75)	11‐876
Number of likes	414 (155.75‐1887.25)	2‐256,000
Number of comments	50.50 (20‐286.25)	0‐81,000
Number of saves	56 (19‐201.75)	0‐21,000
Number of shares	61.50 (12‐275.75)	0‐145,000
Days since upload	382.50 (116.25‐635.75)	21‐1208
Global Quality Score	2 (1‐2)	0‐4
DISCERN score	2.5 (1.63‐3)	0‐3.5

### Uploader Douyin Account Characteristics

Most of the videos in our sample were contributed by professional users (112/136, 82.4%), while a relatively small proportion were contributed by nonprofessional users (24/136, 17.6%). Among professional users, most videos were created by board-certified physicians, followed by hospital channels and health channels (Table 3). Only 1 imaging physician was involved in the posting of 3 videos. The median video duration was significantly longer (*P*<.001) in the nonprofessional group and received significantly more comments (*P*=.004; Table 4).

**Table 3.** Proportion of videos by different types of uploaders.

Source	Description	Videos (n=136), n (%)
Professionals	Individuals or mechanisms who describe themselves as health professionals with certification	112 (82.4)
Board-certified physicians	Medical specialist who diagnoses, treats, and manages diseases and conditions related to breast cancer	106 (77.9)
Health channels	Organizations providing health knowledge	2 (1.6)
Hospital channels	Hospital platforms share health care information	4 (2.9)
Nonprofessionals	Individuals who share mammography experiences or medical personnel without certification	24 (17.6)

**Table 4.** Analysis of video characteristics by source.

Characteristics	Professional (n=112), median (IQR)	Nonprofessional (n=24), median (IQR)	*P* value
Duration (seconds)	46 (31.25‐69.50)	96.50 (53‐132.25)	<.001
Number of likes	382.50 (154‐1658.50)	641.50 (155.75‐15,223)	.33
Number of comments	44 (20‐192.75)	165.50 (42.25‐2900)	.004^a^
Number of saves	55.50 (19.5‐182.5)	75.50 (10.25‐2122.50)	.58
Number of shares	66 (12‐198.75)	59 (11.5‐4561.25)	.57
Days since upload	397 (137‐648.25)	310.5 (78.25‐611.50)	.66
Global Quality Score	2 (1‐2)	1 (1‐1.37)	<.001
DISCERN score	3 (2-3)	0 (0‐0.75)	<.001

a *P*<.01.

As mentioned earlier, the selected videos were divided into useful and unhelpful groups based on scores of the completeness checklist. Of the 136 selected videos, the number of videos containing useful and unhelpful information was 105 (77.2%) and 31 (22.8%), respectively. Notably, despite uniformity in video days since upload between groups, uploads by unhelpful groups garnered more engagement metrics such as likes (median 6892, IQR 585‐104,000), comments (median 1305, IQR 130‐4103), saves (median 748, IQR 53-4381), and shares (median 1056, IQR 50‐6071) relative to useful group, and this differential attains statistical significance (*P*<.01 for all; Table 5). Because the number of nonprofessional uploaders in the useful group was small (7/105), we could not compare this group.

**Table 5.** Analysis of video characteristics by usefulness.

Characteristics	Useful group (n=105), median (IQR)	Unhelpful group (n=31), median (IQR)	*P* value
Duration (seconds)	46 (31-69)	76 (46-114)	<.001
Number of likes	276 (131.50‐815.50)	6892 (585‐104,000)	<.001
Number of comments	35 (17‐113.50)	1305 (130‐4103)	<.001
Number of saves	47 (17-107)	748 (53‐4381)	<.001
Number of shares	48 (10.50‐158)	1056 (50‐6071)	<.001
Days since upload	365 (104.50‐675)	410 (151-538)	.77
Global Quality Score	2 (1.50‐2)	1 (1‐1)	<.001
DISCERN score	2 (2-3)	0 (0‐0)	<.001

### Information Content Comprehensiveness

Useful videos were analyzed based on the information they contained. Among all the categories, the advantages of mammography were the most frequently covered topic (53/105, 50.5%), followed in descending order by applicable objects (50/105, 47.6%), conception (47/105, 44.8%), examination process (44/105, 41.9%), combined other examinations (42/105, 40%), report (26/105, 24.8%), and precautions (11/105, 10.5%; Multimedia Appendix 3). Most of these videos (97/105, 92.4%) scored <5 points, and only 1 video received a maximum score of 7.

### Video Reliability and Quality

The median (IQR) mDISCERN score and GQS of all videos were 2 (1‐2) and 2.5 (1.63‐3), respectively. The Cronbach α coefficients for reliability between the raters were 0.94 and 0.97 for the GQS and mDISCERN, respectively. The mDISCERN score and GQS of the videos in the useful and professional groups were significantly higher than those in the unhelpful and nonprofessional groups (all *P*<.001).

### Correlation Analysis

Spearman correlation analysis revealed certain correlations among the characteristics of the videos. The video duration was positively correlated with the number of comments (*r*=0.23; *P*=.008), saves (*r*=0.20; *P*=.02), and shares (*r*=0.19; *P*=.02). Across all videos, Spearman correlation analysis revealed positive and significant correlations among the number of likes, comments, saves, shares, and days since upload (*P*<.05 for each pair).

The GQS was negatively or positively correlated with the number of likes (*r*=−0.24; *P*=.004), comments (*r*=−0.29; *P*<.001), and saves (*r*=−0.20; *P*=.02) as well as with the mDISCERN score (*r*=0.65; *P*<.001). The mDISCERN score was found to be negatively correlated with the number of likes (*r*=−0.26; *P*=.002), comments (*r*=−0.36; *P*<.001), saves (*r*=−0.22; *P*=.009), and shares (*r*=−0.18; *P*=.03). The correlation coefficients (*r*) reported in this study are generally below 0.39, indicating weak associations. In cases where the correlation coefficients are below 0.1, we consider these to be negligible. We acknowledge that the statistical significance of these correlations may be influenced by the sample size; therefore, we place greater emphasis on the magnitude of the correlation coefficients to better reflect the strength of the relationships. More detailed analytical results are shown in Table 6.

**Table 6.** Correlation analysis (Pearson *r* and 2-tailed *P* value) among the research variables.

Variable	Duration	Likes	Comments	Saves	Shares	Days since upload	GQS^a^	mDISCERN^b^
Duration								
*r* value	1	0.168	0.227^c^	0.201^d^	0.194^d^	–0.088	0.003	–0.144
*P* value	—^e^	.05	.008	.02	.02	.31	.98	.09
Likes								
*r* value	0.168	1	0.909^c^	0.91^c^	0.865^c^	0.284^c^	–0.245^c^	–0.262^c^
*P* value	.05	—^e^	<.001	<.001	<.001	<.001	.004	.002
Comments								
*r* value	0.227^c^	0.909^c^	1	0.851^c^	0.815^c^	0.252^c^	–0.289^c^	–0.361^c^
*P* value	.008	<.001	—^e^	<.001	<.001	.003	<.001	<.001
Saves								
*r* value	0.201^d^	0.91^c^	0.851^c^	1	0.915^d^	0.194^d^	–0.204^d^	–0.222^c^
*P* value	.02	<.001	<.001	—^e^	<.001	.02	.02	.009
Shares								
*r* value	0.194^d^	0.865^c^	0.815^c^	0.915^c^	1	0.353^c^	–0.111^c^	–0.181^d^
*P* value	.02	<.001	<.001	<.001	—^e^	<.001	.20	.03
Days since upload								
*r* value	–0.088	0.284^c^	0.252^c^	0.194^d^	0.353^c^	1	0.087	0.16
*P* value	.31	<.001	.003	.02	<.001	—^e^	.31	.06
GQS								
*r* value	0.003	–0.245^c^	–0.289^c^	–0.204^d^	–0.111	0.087	1	0.651^c^
*P* value	.98	.004	<.001	.02	.20	.31	—^e^	<.001
mDISCERN								
*r* value	–0.144	–0.262^c^	–0.361^c^	–0.222^c^	–0.181^d^	0.16	0.651^c^	1
*P* value	.09	.002	<.001	.009	.03	.06	<.001	—^e^

a GQS: Global Quality Score.

b mDISCERN: modified DISCERN.

c The correlation is significant at a significance level of .05 (2-tailed).

d The correlation is significant at a significance level of .01 (2-tailed).

e Not applicable.

## Discussion

### Principal Findings

This is the first study in the literature to evaluate Douyin content on mammography videos. According to the findings of 2 independent reviewers (C Zhou and HY), more than three-quarters of the videos were uploaded by professional individuals or institutions, and videos containing content primarily concerned with disease knowledge were of higher quality and more reliable. Nevertheless, the overall quality of the mammography videos was poor according to the completeness checklist, GQS, and mDISCERN score. Additionally, the fact that seekers gave higher ratings to the lower-quality videos than the higher-quality videos suggests that most health viewers are not able to identify poor-quality medical information in videos.

The rapid development of digital technology and the widespread application of mobile intelligent terminals have caused various new media to become important platforms for sharing and exchanging scientific knowledge. This has further expanded the channels through which the public can understand and obtain information, broadening the breadth and depth of knowledge. There was an unprecedented reliance on social media platforms to seek information during the COVID-19 pandemic [34]. Douyin is a representative national short video platform, and watching videos every day has become a part of many people’s lives.

Currently, uploaders who share health information on the Douyin app are required to obtain certification materials that verify their affiliation with tertiary A hospitals as doctors. In our study, approximately 80% of mammography-related Douyin contents were uploaded by professional users. Most of them were clinicians; only 1 was an imaging specialist. These findings show that clinicians in tertiary A hospitals with a high level of expertise are enthusiastic about participating in the popularization of mammography-related information. A previous study also showed that radiology-related content on the increasingly popular social media platform TikTok is mainly posted by nonphysician radiology personnel [35]. In addition, our results suggest that the videos cannot cover all aspects of mammography, which may be due to the limited short length of Douyin videos. Furthermore, the most prevalent content of the videos was the advantages of mammography in detecting calcifications; few videos fully addressed other types of content during the examination. This finding may indicate that most publishers believe that the unique advantage of mammography is to help detect breast cancer at an earlier stage. As radiologists, they may be more likely to focus on pain and positioning or precautions during the examination and have a more accurate understanding of diagnostic reports [36]. Pain and discomfort during mammography may influence participation in screening programs and be detrimental to cancer prevention efforts [37]. More senior radiologists should be encouraged to become involved in mammography popularization. Specialized training and publicity should be provided to meet the public’s need for knowledge about mammography.

The current results indicate that the reliability and educational quality of mammography-related videos on Douyin are unsatisfactory, with median mDISCERN and GQS evaluations across all videos stood at 2.5 (IQR 1.63-3), and 2 (IQR 1-2), respectively. This finding is in accord with previous studies that have examined low-quality videos on various health topics and found that this information may not be reliable on Douyin [38,39]. Studies on other video platforms, such as YouTube, also showed that the overall quality of videos providing disease information was poor [40,41]. Because the content of most videos lacks peer or institutional quality review, many may not be subject to quality control and may not be evidence-based; it is therefore not surprising that much of this content is inadequate [42]. Thus, patients should access certified organizations and sites such as those certified with the Health on the Net Foundation Code of Conduct certificate to obtain professional information and avoid being misled by social media. The Health on the Net Foundation Code of Conduct was created as a practical solution to help internet users recognize reliable health-related information on the internet while distinguishing it from potentially erroneous or hazardous content [43]. However, contrary to all these findings, in previous studies of the quality of Douyin videos on children with humeral supracondylar fractures, chronic obstructive pulmonary disease, and cosmetic surgery, the overall information quality and reliability of these short videos were satisfactory in China [44-46]. This might be explained by the assessment instrument’s lack of comparability between different disease categories and the bias introduced by the use of different scoring criteria among different researchers.

The results also showed that videos posted by professionals had significantly higher reliability and GQS than those posted by individuals. This finding indicates that ownership is an important element that can be used to assess the reliability of videos. Video content may be considered trustworthy when produced by professionals such as doctors, medical organizations, and health information websites [47]. Unfortunately, our regression analysis revealed that the number of likes, comments, and saves had a weak negative correlation with both the mDISCERN score and GQS. The results showed that lay users had difficulty distinguishing useful information from a large number of videos. A common misconception is that digital information accuracy is directly related to the number of hits or views [48]. There are thousands of health-related videos promoting misleading information that get millions of views, such as videos that disparage vaccinations [49,50]. These results also indicate that effective regulatory measures are needed to control scientifically accredited information. In the future, it would be beneficial to develop an algorithm that ranks videos preferentially uploaded by a trusted medical center or professional. If the public had less access to unhelpful videos, the damage could be less.

### Limitations

This study has some limitations. First, this was a cross-sectional study that examined a very small portion of a very large amount of data. The number of views, likes, and dislikes of health-related videos on the internet changes over time. The “snapshot” approach to data collection seems to be the main limitation of this study because the results may vary with the use of different search terms and according to the date and time of the search. Second, because of the limitations of the search criteria, it was not possible to include all video resources that fit the topic of this study. Although we included a relatively small percentage of videos, we considered it to be sufficiently representative, as videos beyond the top 100 have no significant impact on the analysis. Third, we only included videos uploaded on Douyin, which is a Chinese video-sharing platform; thus, the findings may not be generalizable to other social media platforms (eg, YouTube) or to other countries. Subsequent cross-linguistic research is required to fill this gap. Finally, the GQS and DISCERN are subjective assessment tools. Although 2 independent experts (C Zhou and HY) determined the ratings iteratively and used Cronbach α coefficients to quantify the agreement between the 2 raters, subjective differences still cannot be ignored. Looking ahead, future research should include broader cross-linguistic comparative studies, using more appropriate assessment instruments to validate our findings.

### Conclusions

According to the findings of our study, a majority of the Douyin videos concerning mammography were uploaded by clinicians and exhibited poor quality and reliability. Patients should not use these videos as the only source of information about mammography because they may lead to misdirected or inappropriate interventions. Douyin is often used to obtain health-related information, and radiologists should be encouraged to provide useful and accurate videos and to instruct patients appropriately. From the standpoint of preventing and curing breast cancer, there is a need for stricter standards and procedures for video publishing to improve the quality of medical content.

## Supplementary material

10.2196/59483 Multimedia Appendix 1 Modified DISCERN criteria.

10.2196/59483 Multimedia Appendix 2 Global Quality Score criteria.

10.2196/59483 Multimedia Appendix 3 Characteristics of useful videos related to each topic.

## Abbreviations

GQS: Global Quality Score
mDISCERN: modified DISCERN
