# Supplementary material for: Evaluation of Douyin Short Videos on Mammography in China: Quality and Reliability Analysis
Source: JMIR Cancer. 2025 Feb 19;11:e59483. doi: 10.2196/59483 (PMC11864564; doi:10.2196/59483)
Supplement: Multimedia Appendix 1 [file cancer-v11-e59483-s001.docx]

| **Reliability of information (1 point for every Yes, 0 points for No)** |
| --- |
| Are the aims clear and achieved? |
| Are reliable sources of information used? (i.e., publication cited, speaker is certified physician) |
| Is the information presented balanced and unbiased? |
| Are additional sources of information listed for patient reference? |
| Are areas of uncertainty mentioned? |

Abbreviation: mDISCERN, modified DISCERN
