# Supplementary material for: Evaluation of Douyin Short Videos on Mammography in China: Quality and Reliability Analysis
Source: JMIR Cancer. 2025 Feb 19;11:e59483. doi: 10.2196/59483 (PMC11864564; doi:10.2196/59483)
Supplement: Multimedia Appendix 2 [file cancer-v11-e59483-s002.docx]

| **Grade** | **Description of quality** |
| --- | --- |
| **1** | Poor quality and unlikely to be of use for patient education |
| **2** | Poor quality and of limited use to patients because some information is present |
| **3** | Suboptimal quality and flow; somewhat useful to patients; important topics are missing; some information is present |
| **4** | Good quality and flow; useful to patients because most important topics are covered |
| **5** | Excellent quality and flow; highly useful to patients |

Abbreviation: GQS, Global Quality Scale
