# Supplementary figures and images for: Evaluation of Douyin Short Videos on Mammography in China: Quality and Reliability Analysis
Source: JMIR Cancer. 2025 Feb 19;11:e59483. doi: 10.2196/59483 (PMC11864564; doi:10.2196/59483)

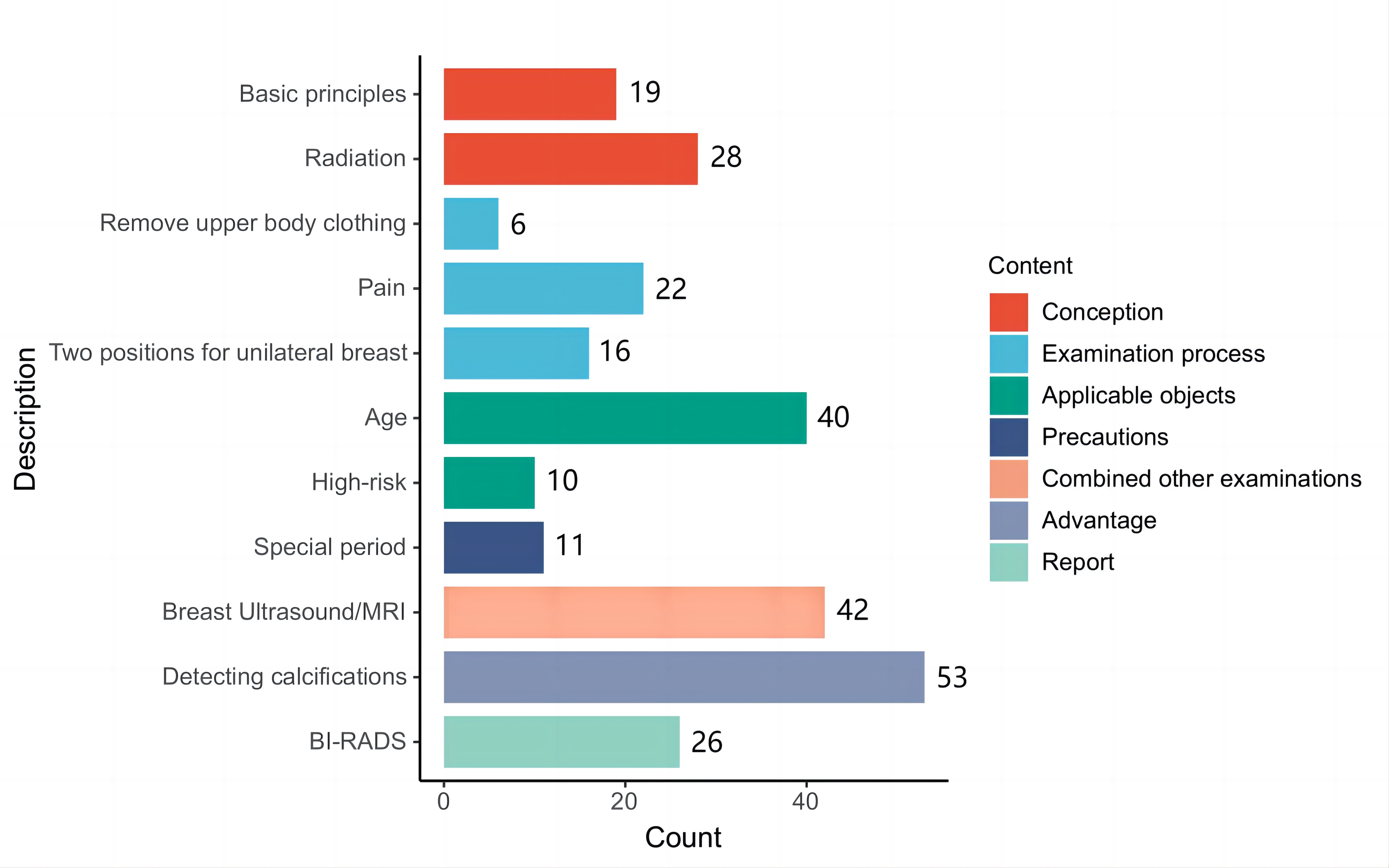

Supplement: Multimedia Appendix 3 [file cancer-v11-e59483-s003.png]
